# Insecticidal activity and biochemical composition of *Citrullus colocynthis*, *Cannabis indica* and *Artemisia argyi* extracts against cabbage aphid (*Brevicoryne brassicae* L.)

**DOI:** 10.1038/s41598-019-57092-5

**Published:** 2020-01-16

**Authors:** Maqsood Ahmed, Qin Peiwen, Zumin Gu, Yuyang Liu, Aatika Sikandar, Dilbar Hussain, Ansar Javeed, Jamil Shafi, Mazher Farid Iqbal, Ran An, Hongxia Guo, Ying Du, Weijing Wang, Yumeng Zhang, Mingshan Ji

**Affiliations:** 1 College of Plant Protection, Shenyang Agricultural University, Shenyang, 110866 P.R. China; 2 College of Biosciences and Biotechnology, Shenyang Agricultural University, Shenyang, 110866 P.R. China; 3 Department of Plant Pathology, University of Agriculture, Faisalabad, Sub-Campus Depalpur, Okara, 56300 Pakistan; 4 Entomological Research Institute, Ayub Agricultural Research Institute, Faisalabad, 38040 Pakistan

**Keywords:** Secondary metabolism, Environmental impact

## Abstract

Plant extracts contain many active compounds, which are tremendously fruitful for plant defence against several insect pests. The prime objectives of the present study were to calculate the extraction yield and to evaluate the leaf extracts of *Citrullus colocynthis* (L.), *Cannabis indica* (L.) and *Artemisia argyi* (L.) against *Brevicoryne brassicae* and to conduct biochemical analysis by gas chromatography-mass spectrometry (GC-MS). The results suggested that when using ethanol, *C. colocynthis* produced a high dry yield (12.45%), followed by that of *C. indica* and *A. argyi*, which were 12.37% and 10.95%, respectively. The toxicity results showed that *A. argyi* was toxic to *B. brassicae* with an LC_50_ of 3.91 mg mL^−1^, followed by the toxicity of *C. colocynthis* and *C. indica*, exhibiting LC_50_ values of 6.26 and 10.04 mg mL^−1^, respectively, which were obtained via a residual assay; with a contact assay, the LC_50_ values of *C. colocynthis*, *C. indica* and *A. argyi* were 0.22 mg mL^−1^, 1.96 and 2.87 mg mL^−1^, respectively. The interaction of plant extracts, concentration and time revealed that the maximum mortality based on a concentration of 20 mg L^−1^ was 55.50%, the time-based mortality was 55% at 72 h of exposure, and the treatment-based mortality was 44.13% for *A. argyi* via the residual assay. On the other hand, the maximum concentration-based mortality was 74.44% at 20 mg mL^−1^, the time-based mortality was 66.38% after 72 h of exposure, and 57.30% treatment-based mortality was afforded by *A. argyi* via the contact assay. The biochemical analysis presented ten constituents in both the *A. argyi* and *C. colocynthis* extracts and twenty in that of *C. indica*, corresponding to 99.80%, 99.99% and 97% of the total extracts, respectively. Moreover, the detected caryophylleneonides (sesquiterpenes), α-bisabolol and dronabinol (Δ^9^-THC) from *C. indica* and erucylamide and octasiloxane hexamethyl from *C. colocynthis* exhibited insecticidal properties, which might be responsible for aphid mortality. However, *A. argyi* was evaluated for the first time against *B. brassicae*. It was concluded that all the plant extracts possessed significant insecticidal properties and could be introduced as botanical insecticides after field evaluations.

## Introduction

The cabbage aphid, *Brevicoryne brassicae* L. (Hemiptera: Aphididae), is a serious pest native to Europe but now found globally^1,2^, and it causes significant losses to economically important crops, including broccoli, oilseed rape, brussels sprouts, cauliflower, black and white mustard, toria, Chinese cabbage, kale, and other field crops^1^. It also causes yellowing, stunting, and plant wilting and acts as a vector of several viral diseases in crucifers, including cauliflower mosaic virus and turnip mosaic virus^3,4^. In the case of severe infestation, plants become covered with aphids, resulting in leaf surfaces roofed with black mould due to honey secretion, which can ultimately cause plant death^5–7^. Synthetic chemical insecticides are available sources for controlling this destructive pest, which is a key success for modern agricultural practices and enhances crop yield. However, indiscriminate use of synthetic pesticides for crop production and protection poses poisonous effects through contact, inhalation, and dietary exposure and has become a cause of carcinogenesis, fertility problems and mutagenesis in humans^8–11^.

These circumstances led towards searching for effective and eco-friendly pest control alternatives, especially from natural plant resources^12^. Many insecticides derived from botanical sources are available and are easily affordable and accessible to the farming community; they are safer for human beings and for the environment with minimal residual effect, and they are target-specific and less toxic to vertebrates, pollinators and fish^13–17^. Studies have demonstrated the aphicidal activity of *Chenopodium ficifolium* extracts and its isolated phospholipids, the alkaloids from *Corydalis turtschaninovii* tubers and *Macleaya cordata* seeds, the oil from *Jatropha curcas* and rhamnolipid from *Pseudomonas* species^18–20^. Other plant-based products, such as *Melia azedarach* seed extracts, *Lantana camorra* leaf and seed extracts and *Mentha piperita* leaf extracts, possess promising insecticidal properties against *B. brassicae*^21–23^.

Therefore, *Citrullus colocynthis* (Cucurbitaceae), *Cannabis indica* (Cannabaceae) and *Artemisia argyi* (Asteraceae) were selected for the present study, as they have been reported as having insecticidal potential against different stored grains and other field crop pests. The selected plants also possess a natural ability to deter insect attack. Among them, *Cannabis* has been reported as a repellent against vertebrates and invertebrates. It also showed better results when grown as a companion crop with other crops to prevent the infestation of weedy plants, fungi, insects and nematodes, while the extract from the dried leaves and flowers possess repellent and killing potential against the above-mentioned pests. However, pure cannabinoids extracted from this plants have been reported to kill or repel mites, insects, nematodes, fungi and protozoa^24^. Moreover, phytoconstituents analysis reported major terpenes, such as β-myrecene and β-caryophyllene, and the profusion of cannabidiolic acid (CBDA) and cannabidiol (CBD) from an extract of *C. sativa* (hemp) inflorescences by GC-MS, GC-FID and HPLC^25^. In a recent study, the activity of hemp essential oil from inflorescences was explored against *Myzus persicae* (Hemiptera), *Culex quinquefasciatus* (Diptera), *Spodoptera littoralis* (Lepidoptera) and *Musca domestica* (Diptera) and showed high toxicity against *M. domestica* and *M. persicae*, moderate toxicity against the larvae of *S. littoralis* and sparse toxicity against *C. quinquefasciatus*^26^.

Similarly, *C. colocynthis* has gained the attention of researchers as an alternative botanical insecticide, and the effectiveness of its extracts and isolated compounds has been assayed against economically important insect pest species. This plant showed antifeedant, deterrent and infertility activities against several pests^27^. The toxicity effects of *C. colocynthis* fruit, leaf, stem and root extracts were evaluated against *Rhopalosiphum padi* L. The results revealed the high effectiveness of the stem extract compared to that of the other part extracts against this pest^28^. Moreover, the identified biocompounds from *C. colocynthis* fruits, i.e., 7,8-benzoquinoline, were the most efficient against *Tetranychus urticae*, while quinoline, 2-methylquinoline and 8-hydroxyquinoline were effective against *Sitophilus zeamais* and *Sitophilus oryzae*^29^.

As a medicinal plant, *A. argyi*, is generally known as Argyi wormwood or Chinese mugwort and is commonly found in China. Different *Artemisia* species are known for their pharmacological and insecticidal properties. The ethanol extracts from *A. argyi* possess a strong repellent effect against *Drosophila melanogaster*, which increased with increasing concentration and extended exposure time^30^. Furthermore, the bioactivity of isolated compounds from *Artemisia judaica* essential oil was evaluated against *Spodoptera littoralis* at the third larval instar, and against plant pathogenic fungi showed prominent insecticidal, antifeedant and antifungal activity. However, the antifeedant activity was concentration-dependent^31^. The antifungal activity of compounds isolated by GC-MS, such as β-thujone, α-thujone, camphor, verbinol and davanone, showed high inhibition effects against *Rhizoctonia solani*, *Fusarium solani*, and *Sclerotinia sclerotiorum* whereas, exhibited slight effectiveness against *Fusarium proliferatum* and *Fusarium oxysporum*^32^.

Due to issues concerning the use of synthetic pesticides and the increasing resistance in pest species, pests can be managed by introducing botanical insecticides, especially against soft-bodied insects such as aphids. However, to our knowledge, few studies have been conducted regarding the aphicidal activity of the leaf extracts of *C. colocynthis*, *C. indica* and *A. argyi* against *B. brassicae*. To investigate safe alternatives for the management of this pest, the present study was conducted to evaluate the insecticidal effects of crude ethanol extracts of *C. colocynthis*, *C. indica* and *A. argyi* against the cabbage aphid *B. brassicae* by residual/leaf dip and contact/aphid dip bioassays under laboratory conditions at the Biopesticides Lab of Shenyang Agricultural University, P.R. China.

## Results

### Extract yield (dry weight %)

The extract yield obtained by solvent extraction using ethanol showed that in comparison, (*P* < 0.05), the extract yield afforded by *C. colocynthis* was 12.45%, followed by that of *A. argyi* and *C. indica* (12.37% and 10.95%) respectively. However, the extract yield produced by all the plant samples was not much different. The extract obtained from *C. colocynthis* and *C. indica* was sticky and fluidic in consistency with a brownish-black to blackish colour and a shiny in appearance. While, the extract from *A. argyi* was waxy in consistency with a yellowish-brown colour.

### Toxicity of plant extracts on aphids

The mortality of *B. brassicae* was determined by using crude ethanol extracts from different plants under laboratory conditions by residual and contact assays. All botanical extracts exerted adverse effects against this pest at increasing concentrations and prolonged exposure periods.

#### Residual/leaf dip bioassay

The mortality data presented in Table S1 in the Supplementary File revealed the insecticidal activity of ethanol extracts from *C. colocynthis*, *C. indica* and *A. argyi* against *B. brassicae*. The percent mortality of *B. brassicae* was directly related to the concentration of the plant extracts and the exposure period. The results showed that the maximum mortality was recorded after 72 h of exposure to the *A. argyi* extract at a concentration of 20 mg mL^−1^ and caused 88.33 ± 3.87% mortality, while after 48 h of exposure, the mortality was recorded as 60.00 ± 2.27%. The mortality caused by *C. indica* after a 72 h and 48 h exposure period was 66.67 ± 2.58% and 45.00 ± 4.72%, respectively, which were lower than those of *A. argyi*. However, the mortality caused by *C. colocynthis* was higher than that caused by *C. indica* after a 72 h exposure period (76.67 ± 1.29%) but decreased to 40.00 ± 2.23% after 48 h of exposure at the same concentration. The mortality of *B. brassicae* by *A. argyi* (35.00 ± 3.87%) was also higher after 24 h of exposure than that of 24 h of exposure to *C. colocynthis* and *C. indica* extracts (23.33 ± 2.58% and 20.00 ± 2.23%, respectively). The control treatment showed no mortality after 24 h and 48 h and negligible mortality (1.67 ± 1.29%) after 72 h, whereas imidacloprid was the positive control and showed the highest (*P* < 0.05) mortality, i.e., 98.33 ± 2.58, 91.67 ± 2.58 and 86.67 ± 1.29% after 72 h, 48 h and 24 h, respectively.

The results described in Table S2 in the Supplementary File revealed highly significant (*P* < 0.001) model fitness with (*F* = 101.057, 9851.888, 59.047, 754.712 and 545.650) intercept, concentration and time. Moreover, the interaction between the mortality (%) of the aphids versus time and concentration exhibited a highly significant (*P* < 0.001) positive correlation (*F* = 8.62). The two-way analysis of variance provided information regarding the interaction between mortality (%), treatment and time and highlighted highly significant results (*F* = 7.25). Interaction/correlation between treatments × concentration × time and the total variance was firmly trait-specific and significantly correlated with mortality (*P* < 0.001 and with *F* = 2.75).

However, data for interaction among plant species, concentration and post-treatment time revealed that the maximum mean mortality based on concentration by different treatments/plant species was recorded at 20 mg mL^−1^, with 50.56 ± 7.92% followed by 43.52 ± 7.52, 35.37 ± 7.02, 28.89 ± 6.12% and 20.19 ± 3.16% at 15, 10, 5 and 2.5 mg mL^−1^, respectively. The maximum mean mortality interaction based on time was recorded as 55.00 ± 6.79% at 72 h exposure, followed by 36.35 ± 6.02 and 23.89 ± 5.79% at 48 and 24 h, respectively. On the other hand, mortality interaction among treatments was evaluated as high (44.13 ± 7.14%) by *A. argyi* followed by that of *C. indica* and *C. colocynthis* (36.99 ± 6.67% and 34.13 ± 6.53%, respectively). Moreover, the positive control showed a maximum 90.19 ± 2.09% mortality, and the negative control showed a minimum mortality of 0.19 ± 0.18%. Probit analysis showed the LC_50_ values, slope, chi-square, and fiducial limits at the 95% confidence interval. Although *B. brassicae* showed sensitivity to the different plant extracts, *A. argyi* was the most toxic, followed by *C. colocynthis* and *C. indica*, and this mortality response is presented in Table 1.

**Table 1:** Toxicity against *B. brassicae* after an exposure of 24, 48 and 72 h by the residual/leaf dip method.

Plants Extract	Time (h)	LC_50_ (mg mL^−1^)	95% F.L.		Slope ± SE	χ^2^
			Lower	Upper		
CCL	24	466	64.1	1.E20	1.34 ± 0.29	0.17
	48	68.1	28.3	340.7	0.76 ± 0.25	1.73
	72	10.4	7.61	13.53	1.44 ± 0.26	6.52
CIL	24	273	59.4	597.7	0.76 ± 0.31	0.13
	48	36.4	18.8	414.0	0.78 ± 0.25	1.08
	72	6.26	2.60	10.37	0.75 ± 0.23	0.09
ATL	24	38.6	24.4	114.5	1.31 ± 0.29	1.16
	48	13.8	10.1	21.9	1.20 ± 0.25	1.03
	72	3.91	2.66	5.07	1.67 ± 0.26	1.80

Note: LC_50_ (lethal concentrations); S.E. (standard error); χ^2^ (chi-square); F.L. (fiducial limit). *upper limits are greater than or equal to 1.E20 and are infinite values. CCL (*C. colocynthis*), CIL (*C. indica*) and ATL (*A. argyi*).

#### Contact/aphid dip bioassay

The data presented in Table S3 in the Supplementary File described the mortality (%) of *B. brassicae* by the contact toxicity method. The results showed that maximum mortality was recorded after 72 h at 20 mg mL^−1^. However, the highest mortality caused by the crude extract of *A. argyi* was 93.33 ± 3.14%, followed by that of *C. colocynthis* and *C. indica* (83.33 ± 2.57 and 81.67 ± 1.29%, respectively). High mortality after 48 h of exposure to 15 mg mL^−1^ was also observed in *A. argyi* (83.33 ± 1.29%), followed by that of *C. colocynthis* and *C. indica*, which had the same mortality values of 81.67 ± 3.41% and 81.67 ± 1.67%, respectively. Additionally, the mortality of *B. brassicae* by the contact assay was also high for exposure to extracts of *C. indica* even at low concentrations (78.33 ± 2.58%, 71.67 ± 3.41% and 71.67 ± 3.41% at concentrations of 10, 5 and 2.5 mg mL^−1^, respectively). The same trend of mortality was observed for *C. colocynthis* and *A. argyi*. The negative control treatment showed similar results for mortality in all three experiments for each treatment in both bioassay methods, while imidacloprid showed significantly (*P* < 0.05) high mortality (98.33 ± 1.29%, 96.67 ± 2.58% and 95.00 ± 2.23% after 72 h, 48 h and 24 h, respectively) for leaf dip and contact bioassay, which was also the highest mortality.

The results described in Table S4 in the Supplementary File revealed also highly significant (*P* < 0.001) model fitness with (*F* = 44.42, 11152.07, 14.66, 409.34 and 67.44) intercept, concentration and time. However, the interaction between mortality (%) of the aphid versus time and concentration recorded a highly significant (*P* < 0.001) positive correlation (*F* = 1.58). The two-way analysis of variance provided information regarding the interaction between mortality (%) versus treatment and time highlighted highly significant results (*F* = 10.07). The interaction/correlation between treatments × concentration × time and total variance was strongly trait-specific and highlighted significant correlation with mortality (*P* < 0.001 with *F* = 1.79). However, data for the interaction among plant species, concentrations and post-treatment time revealed that the maximum mean mortality based on concentration by different treatments/plant species was recorded at 20 mg mL^−1^, with 74.44 ± 3.74% followed by 70 ± 3.58, 63.33 ± 3.08, 56.48 ± 4.34 and 47.59 ± 4.74% at 15, 10, 5 and 2.5 mg mL^−1^, respectively. The maximum mean mortality interaction based on time was recorded as 66.83 ± 6.63% after 72 h of exposure, followed by 56.27 ± 6.19% and 51.51 ± 6.82% after 48 and 24 h, respectively. The mortality interaction among treatments was 57.30 ± 6.31% by *A. argyi* followed by 62.22 ± 7.14% and 55.08 ± 6.62% by *C. colocynthis* and *C. indica*, respectively. Moreover, the positive control treatment showed a maximum of 95 ± 1.14% mortality, and a 0.56 ± 0.27% mortality was exhibited by the negative control.

Probit analysis of the data revealed the LC_50_ values, slope, chi-square, and fiducial limits at the 95% confidence interval for *C. colocynthis*, *C. indica* and *A. argyi*. Mortality response using different plant extracts showed high sensitivity of *B. brassicae* to *A. argyi* followed by *C. colocynthis* and *C. indica* in the contact assay, which is summarized in Table 2.

**Table 2:** Toxicity against *B. brassicae* after an exposure of 24, 48 and 72 hours by the contact/aphid dip method.

Plants Extract	Time (h)	LC_50_ (mg mL^−1^)	95% F.L.		Slope ± SE	χ^2^
			Lower	Upper		
CCL	24	2.88	0.03	4.29	0.59 ± 0.23	0.51
	48	2.35	0.62	4.01	1.01 ± 0.26	0.77
	72	1.96	0.14	3.88	0.72 ± 0.24	0.80
CIL	24	12.1	8.86	19.3	1.07 ± 0.23	0.06
	48	8.03	4.37	13.8	0.79 ± 0.24	0.53
	72	2.87	0.10	5.75	0.65 ± 0.25	0.14
ATL	24	5.62	2.31	8.91	0.72 ± 0.22	0.98
	48	4.28	1.09	7.13	0.72 ± 0.23	0.38
	72	0.22	0.01	1.31	0.58 ± 0.27	0.30

Note: LC_50_ (Lethal concentration); S.E. (standard error); χ^2^ (chi-square); F.L. (fiducial limit). (*C. colocynthis*), CIL (*C. indica*) and ATL (*A. argyi*).

### Gas chromatography-mass spectrophotometry (GC-MS) analysis

The presence of biologically active components from the ethanol extracts of *C. colocynthis*, *C. indica* and *A. argyi* were evaluated by conducting GC-MS analysis. The principal active compounds, molecular weight (g mol^−1^, M.W.), molecular formula (M.F.), retention time (R.T.) and peak area (%) are presented in Tables 3–5. The results of GC-MS analysis of the extracts led to the determination of several biological compounds.

**Table 3:** Chemical composition of the *C. colocynthis* extract.

Peak. No.	RT	Compounds	Area %	M.F.	M.W.
1	3.16	Cyclotrisiloxane, hexamethyl-	3.57	C_6_H_18_O_3_Si_3_	222.46
2	11.9	Triamterene	1.53	C_12_H_11_N_7_	253.26
3	14.6	Palmitic acid, ethyl ester (Hexadecanoic acid)	0.98	C_18_H_36_O_2_	284.47
4	16.3	Octasiloxane,1,1,3,3,5,5,7,7,9,9,11,11,13,13,15,15-hexadecamethyl-	1.25	C_16_H_50_O_7_Si_8_	579.24
5	16.9	Tetrasiloxane, decamethyl-	1.10	C_10_H_30_O_3_Si_4_	310.68
6	22.6	Octasiloxane,1,1,3,3,5,5,7,7,9,9,11,11,13,13,15,15-hexadecamethyl	7.06	C_16_H_50_O_7_Si_8_	579.24
7	24.7	Heptasiloxane, 1,1,3,3,5,5,7,7,9,9,11,11,13,13-tetradecamethyl-	21.3	C_14_H_44_O_6_Si_7_	505.09
8	25.6	5-Methyl-2-phenylindolizine	17.9	C_15_H_13_N	207.27
9	25.8	Erucylamide	22.4	C_18_H_35_NO	337.58
10	26.6	4-Nitro-4′-chlorodiphenylsulfoxide	22.9	C_12_H_8_ClNO_2_S	265.71

M.F. (molecular formula); M.W. (molecular weight); RT (retention time).

**Table 4:** Chemical composition of the *C. indica* extract.

Peak No	RT	Compounds	Area %	M.F.	M.W.
1	9.91	Caryophyllene oxide	0.70	C_15_H_24_O	220.35
2	10.2	3-Methoxybenzyl alcohol	0.39	CH_3_O_6_H_4_CH_2_OH	138.16
3	10.7	5H-Naphtho[1,8-bc]thiophen-5-one,3,4-dihydro-2-methyl-	1.20	C_12_H_10_OS	202.27
4	10.9	α-Bisabolol	0.66	C_15_H_26_O	222.37
5	11.9	2,5-Cyclohexadien-1-one, 2,5-dimethyl-4-[(2,4,5-trimethylphenyl) imino]-	0.41	C_17_H_19_NO	253.34
6	16.2	Phytol	1.51	C_20_H_40_O	296.30
7	16.8	1,3,4-Trimethoxydibenzofuran	0.29	C_15_H_14_O_6_	290.07
8	17.1	9,12,15-Octadecatrienoic acid, ethyl ester, (Z,Z,Z)-	0.36	C_20_H3_4_O_2_	306.49
9	18.1	2,11-Dimethyl-2,3,4,5,6,7-hexahydro-1H-2-benzazonine	1.04	C_14_H_24_N	203.32
10	18.9	Acridine	0.47	C_13_H_9_N	179.13
11	19.3	Benzenesulfonic acid, 4-nitro-	25.5	C_6_H_5_NO_5_S	213.17
12	19.4	1H-Indene-4-acetic acid, 6-(1,1-dimethylethyl)-2,3-dihydro-1,1-dimethyl- methyl ester	1.20	C_18_H_26_O_2_	274.40
13	20.1	2H-1-Benzopyran-5-ol, 2-methyl-2-(4-methyl-3-penten-1-yl)-7pentyl- (Cannabichromene)	0.53	C_21_H_30_O_2_	314.46
14	20.2	6H-Dibenzo[b,d]pyran-1-ol, 6,6,9-trimethyl-3-propyl- (Cannabivarin)	1.00	C_19_H_22_O_2_	282.38
15	20.9	Resorcinol, 2-p-mentha-1,8-dien-3-yl-5-pentyl-, (−)-(E)-	0.53	C_21_H_30_O_2_	314.47
16	20.9	2H-1-Benzopyran-5-ol, 2-methyl-2-(4-methyl-3-pentenyl)-7pentyl-, (+/−)-(Cannabichromene)	2.00	C_21_H_30_O_2_	314.47
17	21.7	Cyclobarbital	2.19	C_12_H_16_N_2_O_3_	236.27
18	22.3	Dronabinol	57.3	C_21_H_30_O_2_	314.45
19	23.1	1,3-Benzenediol, 2-(3,7-dimethyl-2,6-octadienyl)-5-pentyl-	0.40	C_21_H_32_O_2_	316.47
20	23.3	Cannabinol	2.29	C_21_H_26_O_2_	310.43

M.F. (molecular formula); M.W. (molecular weight); RT (retention time).

**Table 5:** Chemical composition of the *A. argyi* extract.

Peak No	RT	Compounds	Area %	M.F.	M.W.
1	3.49	1,5-Heptadien-4-ol, 3,3,6-trimethyl-	3.46	C_10_H_18_O	154.24
2	10.2	1,2-Ethanediol, diformate	1.44	C_4_H_6_O_4_	118.08
3	10.5	Neodecanoic acid	43.6	C_10_H_20_O_2_	172.26
4	10.7	2-Trimethylsilyl-1,3-dithiane	21.6	C_17_H_16_S_2_Si	192.42
5	16.4	N-Isopropyl-3-phenylpropanamide	4.37	C_12_H_17_NO	191.27
6	16.8	2-Isopropenyl-2,3-dihydrofuro[3,2-g]chromen-7-one	6.28	C_14_H_12_O_3_	228.25
7	21.7	1-Propanone, 3-(2-hydroxyphenyl)-1,3-diphenyl-	2.00	C_21_H_18_O_2_	302.37
8	22.9	2-Ethylacridine	3.71	C_15_H_13_N	207.27
9	23.3	5-Methyl-2-phenylindolizine	2.81	C_15_H_13_N	207.27
10	25.7	Cyclotrisiloxane, hexamethyl-	10.5	C_6_H_18_O_3_Si_3_	222.46

M.F. (molecular formula); M.W. (molecular weight); RT (Retention time).

The crude extract of *C. colocynthis* leaves showed the presence of ten compounds corresponding to 99.99% of the total extract; however, 4-nitro-4′-chlorodiphenylsulfoxide (22.94%), erucylamide (22.38%), heptasiloxane,1,1,3,3,5,5,7,7,9,9,11,11,13,13-tetradecamethyl- (21.33%), 5-methyl-2-phenylindolizine (17.85%), and octasiloxane,1,1,3,3,5,5,7,7,9,9,11,11,13,13,15,15-hexadecamethyl- (7.06%) were the major compounds, and other minor compounds were present in low quantities, with relative peak areas ranging from 0.98–3.57%.

However, the results observed through GC-MS analysis of *C. indica* revealed the presence of twenty compounds corresponding to 97% of the total extract. Dronabinol (57.29%), benzenesulfonic acid, 4-nitro- (25.54%), cannabinol (2.29%), and cyclobarbital (2.19%) were the main compounds, while sixteen other minor compounds were reported, with relative peak areas ranging from 0.29–2.00%.

The chemical profiling obtained through GC-MS analysis of *A. argyi* revealed the presence of ten compounds corresponding to 99.80% of the total extract; however, neodecanoic acid (43.60%), 2-trimethylsilyl-1,3-dithiane (21.61%), cyclotrisiloxane, hexamethyl- (10.62%), 2-isopropenyl-2,3-dihydrofuro[3,2-g]chromen-7-one (6.28%), and N-isopropyl-3-phenylpropanamide (4.37%) were the major compounds, and five other minor compounds with lower relative peak areas ranging from 1.44–3.81% were identified.

## Discussion

Due to the problems concerning the use of synthetic chemicals for pest management, there is an urgent need to introduce natural products, mainly from plant sources, for use against insect pests, especially *B. brassicae*, which is becoming problematic in cabbage growing areas of the world. Plant extracts or essential oils are commonly applied for pest control measures because of their effectiveness against different life stages of the pests. However, the selected plants (*C. colocynthis*, *C. indica* and *A. argyi*) are naturally repellent to some extent to insect attack, which indicates that they could be suitable candidates against cabbage aphids, *B. brassicae*. An investigation was performed to detect the phytochemical constituents of the extracts of the above-mentioned plants, and their insecticidal activity was evaluated by residual and contact toxicity methods because of the feeding style of aphids^33^.

However, compound extraction from plant parts depends upon the type of plant material and solvent used. High-polarity solvents produced a higher yield than that of low-polarity solvents. In our results, ethanol afforded high extract yields during solvent extraction, which was due to its high polarity. Previously, it was reported that ethanol yielded more than other solvents from leaves of *Melastoma malabathricum*^34^. Furthermore, a relatively high extract yield from *C. colocynthis* and *C. sativa* using ethanol as an extraction solvent was reported^35^.

According to our findings, the mortality of *B. brassicae* using plant extracts increased with increasing concentrations and exposure periods. However, the physical and chemical properties of the essential oils exhibited different persistent levels of insecticidal properties and different action mechanisms^36,37^. It was reported that extracts of *Cassia sophera* and *Ageratum conyzoides* against *B. brassicae* induced mortality that was equivalent to that of the positive control, the synthetic insecticide emamectin benzoate^38^. Moreover, a *Mentha piperita* extract resulted in maximum insecticidal activity against *B. brassicae* with increased concentration and time exposure^39^. Similarly, essential oil from *Cinnamomum zeylannicum* was found to be effective against *B. brassicae*, exhibiting 8.3 and 7.4 µl mL^−1^ LC_50_ values^40^. The essential oil of another medicinal plant, *Elettaria cardamomum*, was reported to be effective against this serious pest, exhibiting 79.2 µl mL^−1^ LC_50_ value^41^. There are reports that *B. brassicae* was sensitive to 1,8 cineole, a chemical compound found in *Laurus nobilis*, which at LD_50_ value of 30 µl mL^−1^ caused 52% mortality^42^. Additionally, essential oils isolated from natural plants are usually considered as safe for humans and environment which can be a source of new botanical insecticides. Among some of the plant species, *Ocimum basilicum*, *Mentha piperita*, *Pimpinella anisum, Mentha pulegium* and *Foeniculum vulgare* have shown outstanding effectiveness against aphids species in both contact and fumigation assays and can thus be considered as active materials for developing new botanical agents^43^. It was also reported that the use of *Azadirachta indica* oil as a suitable alternative to synthetic chemicals against *B. brassicae* caused a noticeable reduction in their population^44^. All these findings are consistent with our results on the mortality of *B. brassicae* at different concentrations and varying levels of LC_50_ values.

The results of the studies using the *C. colocynthis* leaf extract to control *B. brassicae* are exceptional. However, toxicity of *C. colocynthis* extracts from leaves and fruits against *Aphis craccivora* was reported^45^. Cucurbitacin E glycosides isolated from *C. colocynthis* extract showed strong insecticidal effects against *Aphis craccivora*^46^. Limonene contained by *Citrus sinensis* was reported to be effective against *Myzus persicae* at LC_50_ 57.7 µl mL^−1^, whereas its essential oil at a concentration of 3.3 µl mL^−1^ caused significant mortality of *B. brassicae*^41^. All these results support our findings of using *C. colocynthis* extract against *B. brassicae*. However, according to our results from the GC-MS analysis, the chemical composition of *C. colocynthis* was dominated by four of the ten detected compounds. Among these, erucylamide is a high fatty acid amide that can be used as an insecticide, pigment dispersant, ink and paint additive, fibre softener, and in dyes. Octasiloxane hexamethyl from *C. colocynthis* also exhibits insecticidal properties.

The insecticidal activity of the essential oil of hemp inflorescences against *M. persicae* was reported to be highly toxic, with an LC_50_ of 3.5 mL L^−1^ and non-toxic to the beneficial organisms^26^. An important compound, (E)-caryophyllene myrecene, and α-pinene, contained by *C. sativa*, showed 98.20% mortality at 3.50 µl mL^−1^ of *Aulacorthum solani*^47^, whereas at the same concentration, similar mortality of *M. persicae* was reported^26^. Previous studies have reported the concentration and time-dependent mortality of *Tetranychus urticae* and *Aulacorthum solani* following the use of hemp essential oil, which supported our findings indicating the effectiveness of the hemp (*C. sativa*) crude extract against *B. brassicae* aphids^47^. However, among the twenty compounds identified in the crude ethanol extract, caryophylleneonides (sesquiterpenes), α-bisabolol and dronabinol (Δ^9^-THC) possess insecticidal activities, However, caryophyllene oxide identified from *Melaleuca styphelioides* exhibited strong aphicidal activities against *Aphis spiraecola*, *Aphis gossypii* and *M. persicae*^48^ while, the other compounds may need to be further explored to determine their biological properties. To date, few studies on the applicability of hemp essential oil against crop pests have been conducted, and the use of hemp essential oil against *B. brassicae* is very rare in the literature.

Similarly, *A. argyi* is a famous plant commonly found in China, and many species of the genus *Artemisia* have long been used in traditional Chinese medicines, including *Artemisia argyi*, *Artemisia capillaris* and *Artemisia annua*^49^. In the present study, the crude ethanol extract from the leaves of *A. argyi* was proven to be highly toxic against *B. brassicae*. Moreover, four compounds from *A. argyi*, such as camphor, β-pinene, eucalyptol, and β-caryophyllene, showed strong toxic effects against adults of *Lasioderma serricorne*^50^. Other studies have showed that sabinene, and β-myrecen contained in *Artemisia absinthium*, caused significant mortality of *M. persicae* at 6.9 µl mL^−1^ concentration^51^. Similarly, *Artemisia seiberi* essential oil caused significant mortality of *Eriosoma lanigerum* at 6.16 µl mL^−1^ LD_50_ value^52^. Another compound, artemisia ketone, showed significant mortality of *B. brassicae* at 25.0 µl mL^−1^ concentration^53^. However, according to our results, reported compounds from *A. argyi* leaves were evaluated for the first time for their insecticidal potential. The above-described results supported our findings and clearly demonstrated that the *A. argyi* crude extract exhibited strong and deadly effects against *B. brassicae*, which justified its great potential for controlling this pest.

In the present study, the toxicity of ethanol extracts of the leaves of *C. colocynthis*, *C. indica*, and *A. argyi* against *B. brassicae* were investigated under laboratory conditions, revealing their positive toxicity. *A. argyi* proved to be the most toxic in both bioassay methods and caused maximum mortality, followed by *C. colocynthis* and *C. indica*, in a dose-dependent manner. This insecticidal activity might be due to individual efficacy or the synergistic action of biological compounds present in these plants. A literature survey regarding the potential efficacy of *A. argyi* showed that there are no studies reporting on its use against *B. brassicae*. Additionally, data on the insecticidal potential of *C. colocynthis* and *C. indica* against this serious pest and their GC-MS profiling are limited to a few studies. Thus, the present unique and novel study was conducted for the first time to investigate the insecticidal potential of crude ethanol extracts by residual and contact toxicity methods against *B. brassicae*, a serious pest of cabbage and other field crops.

## Conclusions

The investigations indicated that *C. colocynthis*, *C. indica* and *A. argyi* possess potential botanical agents as an alternative to synthetic pesticides against *B. brassicae*. It can be concluded that, *B. brassicae*, is reasonably sensitive to *A. argyi* followed by *C. colocynthis* and *C. indica* as shown in both bioassay methods, even at low concentrations and exposure times. Moreover, in comparison, the contact assay afforded higher mortality than that of the residual assay. Additionally, GC-MS analysis revealed the presence of valuable biologically active compounds responsible for aphid mortality. However, further studies are required on the purification and identification of active components responsible for aphid mortality and their evaluations against other insect pests.

## Materials and Methods

### Collection of plant material

Aerial parts of the plants tend to contain a wider variety of compounds, and most of the photosynthesis/respiration occurs in the aerial parts. Additionally, most of the secondary metabolites from plants are produced in leaves. For this reason, *C. colocynthis* (Colocynthis) leaves at the 15–20 leaf stage were collected from Bahawalnagar (29°59′34″N, 73°15′13″E) Punjab, Pakistan from the natural habitat of a desert climate from March to April 2018 and authenticated at the Entomological Research Institute, Faisalabad, Pakistan. This area contains a dry climate with average annual precipitation of 204 mm and a temperature range of 12.7–45 °C. *C. indica* (wild hemp) and *A. argyi* (Chinese mugwort) leaves (10–15 leaf stage) were collected from Shenyang (41°11′43″N, 122°25′48″E) Liaoning, China, from a temperate climate zone from May to June 2018. This area receives annual precipitation of 266.6 mm, and the temperature ranges from −28.5 to 36.1 °C. The collected samples were authenticated at the College of Plant Protection, Department of Pesticides Science, Shenyang Agricultural University, Liaoning P.R., China.

### Preparation of plant material and extraction

The collected leaves were allowed to dry in the shade at room temperature after removing impurities by rinsing in tap water. Dried leaves were ground into a fine powder using an electric blender, and 99.7% ethanol was used as a solvent for extraction. Approximately 100 g of ground powder was extracted thrice with 400 mL of ethanol each time for 72 h at a constant temperature in an incubator shaker (ZWY-1102C) at 100 rpm. After each extraction, the material was filtered through filter paper (Whatman No. 1) and concentrated to reduce the volume by using a rotary evaporator (Buchi Switzerland R-210). Finally, the concentrated filtrate was subjected to drying in a fume hood at 25 °C for 12 h. The extraction yield was measured for each extract, and the extracts of each plant were combined to obtain a bulk sample and stored in glass-stoppered vials at 4 °C until use.

### Collection and breeding of aphids

Aphids, *B. brassicae*, were collected from a cabbage field (without pesticidal application) in the experimental field area of Shenyang Agricultural University, China. Aphids were reared on cabbage plants in the lab under controlled conditions of 20 ± 5 °C and 45 ± 5% R.H., along with a photoperiod of 16:8 (light: dark).

### Serial concentration and agar preparation

Tween-20 was used to minimize variations among the treatments effect of any adjuvant material. Fifty millilitres of Tween was dissolved in 950 mL distilled water by shaking to prepare a 5% solution. Serial concentrations of crude extract, 2.5, 5, 10, 15 and 20 mg mL^−1^ were prepared in the Tween solution one day before beginning the experiment. To prepare the agar, 10–15 g agar rods were added to one litre of distilled water, boiled for 10 min with continuous stirring, allowed to cool for 10 min and was then added to Petri dishes 90 mm in diameter and 15 mm deep up to a 3–4 mm level.

### Bioassay study

Two different modes of exposure, residual and contact assays were conducted for the toxicity evaluation. The experiment was replicated five times for each exposure method as well as concentration, and the mortality response was recorded at the exposure times of 24 h, 48 h and 72 h.

### Residual toxicity/leaf dip bioassay

**Figure 1:**
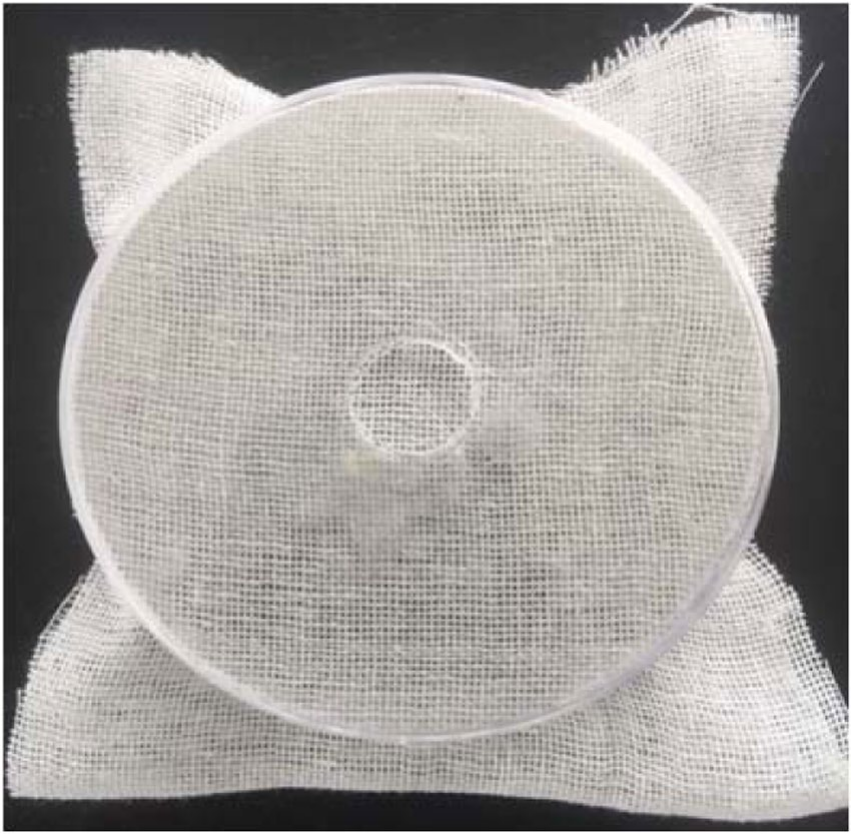
Petri dishes utilized for residual bioassay.

Briefly, cabbage leaf discs 5 cm in diameter were cut with sterilized scissors and dipped for 10 s in the extracts of varying concentration. Then, the leaf discs were left at room temperature for 10 min to dry and put in Petri dishes containing agar. Then, twenty aphids at the second instar were carefully transferred onto the leaf disc contained in the Petri dish by a fine-haired brush, and caution was taken to prevent injury to the aphids during their transfer to the leaf disc. Then, a double layer of muslin cloth was placed on each Petri dish and covered with a manually perforated lid to avoid suffocation and escape of the aphids (Fig. 1). The negative control treatment was prepared using a Tween-20 (5%) solution as described above but without added plant extract. An imidacloprid solution  of 20% SL (2.5 mL L^−1^) was used as a positive control. All Petri dishes were incubated for 72 h at 65% R.H., 25 °C and with a 16:8 (light: dark) photoperiod.

### Contact toxicity/aphid dip bioassay

**Figure 2:**
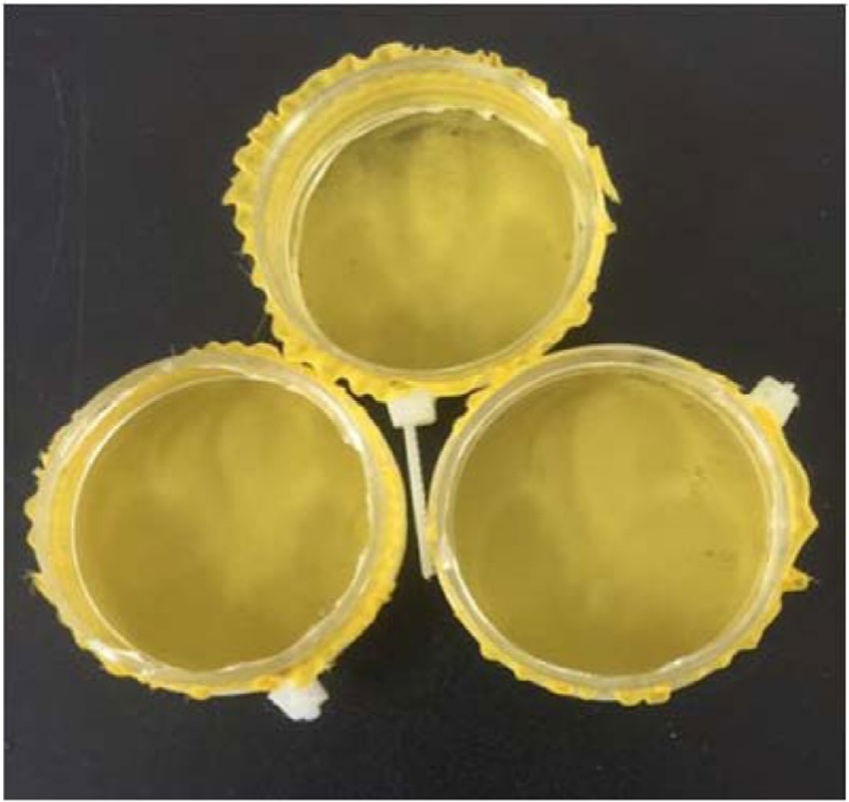
Containers utilized for contact bioassay.

A 30 mm diameter plastic Petri dish was cut from the basal portion, and the remaining ring was used as the container. A nematode strainer (mesh size 300 µm) was used as a holding cell for aphids during dipping into the plant extracts of varying concentration (Fig. 2). Then, twenty aphids at second instar were isolated from the cabbage plants and dipped in the extracts for 10 s. Each group of dipped aphids was then placed on fresh 5 cm diameter cut leaf discs on agar-containing Petri dishes. The negative control treatment was prepared using the Tween-20 (5%) solution as described above but without adding plant extract. An imidacloprid solution of 20% SL (2.5 mL L^−1^) was used as a positive control. All Petri dishes were incubated for 72 h at 65% R.H., 25 °C and with a 16:8 (light: dark) photoperiod.

### Data collection

Mortality data were recorded at the exposure times of 24 h, 48 h and 72 h by microscopic examination. The response of *B. brassicae* to the crude extracts was observed by needle stimulation, and those individuals who displayed no response were considered dead.

### Sample preparation for GC-MS analysis

The solvent-free extract was weighed precisely to 0.005 g and dissolved in 1 ml of HPLC-grade methanol. Then, the sample was added to a centrifuge tube, and graphitized carbon blacks (GCB) was added (0.002 mg/tube) and shaken vigorously for one min on a vortex (Vortex Gennic 2, Model G560E Scientific Industries Inc. U.S. A) to remove coloured components. The colourless sample was centrifuged for 10 min at 24 °C and 12000 rpm (Hitachi Centrifuge CT15RE, Koki Co., Ltd. Japan) to separate the GCB from the sample. The colourless sample was collected by micropipette and transferred to a new centrifuge tube.

### Gas chromatography-mass spectrophotometry (GC-MS) analysis

The crude ethanol extracts of *C. colocynthis*, *C. indica* and *A. argyi* were analysed via GC-MS using an Agilent 6890–5973N USA gas chromatograph (GC) equipped with an HP1 TG-5MS polydimethylsiloxane capillary column (30 m × 250 µm × 0.25 µm) interfaced with a Hewlett Packard 5973 mass selective detector. Gas chromatographic parameters were as follows: the temperature was fixed at 110 °C for 2 min initially and increased to 200 °C and 280 °C at increasing rates of 10 °C min^−1^ and 5 °C min^−1^, respectively. The inlet temperature was 250 °C; a 10:1 split ratio was used; MS quadrupole temperature, 150 °C; thermal aux temperature, 285 °C; ionization current, 60 µA; MS temperature, 230 °C; MS scan range, m/z 40–450; ionization energy, 70 eV; carrier gas, helium with flow rate, 1.0 mL min^−1^. Compounds were identified by comparison of the GC-MS mass spectra with those in the literature or in databases at Wiley/NIST.98.1^54,55^. The relative abundance of each compound was assessed based on the raw data peak areas in the spectra with no response factor correction from the FID.

### Data analysis

All recorded mortality data were subjected to two-way analysis of variance (ANOVA) for calculating the interaction among plants, concentration and post-treatment time, while the mean difference between treatments was calculated for the significance test by Tukey’s HSD at the *P* = 0.05 level. All statistical processes were administered by different statistical packages with IBM-SPSS version 25.0 software, while LC_50_ values, slope, chi-square values and fiducial limits were calculated by the EPA Probit analysis program version 1.5.

## Supplementary information

supplementary information.
