# Supplementary material for: Insecticidal activity and biochemical composition of Citrullus colocynthis, Cannabis indica and Artemisia argyi extracts against cabbage aphid (Brevicoryne brassicae L.)
Source: Sci Rep. 2020 Jan 16;10:522. doi: 10.1038/s41598-019-57092-5 (PMC6965137; doi:10.1038/s41598-019-57092-5)
Supplement: Supplementary file 1 — supplementary information. [file 41598_2019_57092_MOESM1_ESM.pdf]

Insecticidal activity and biochemical composition of *Citrullus colocynthis*, *Cannabis indica* and *Artemisia argyi* extracts against cabbage aphid (*Brevicoryne brassicae* L.)

Maqsood Ahmed[1,+], Qin Peiwen[1,*], Zumin Gu[1], Yuyang Liu[1], Aatika Sikandar[1,+], Dilbar Hussain[4], Ansar Javeed[2,+], Jamil Shafi[3], Mazher Farid Iqbal[2], Ran An[1], Hongxia Guo[1], Ying Du[1], Weijing Wang[1] , Yumeng Zhang[1] & Mingshan Ji[1,*]

Supplementary Tables.

Table S 1. Mean mortality of *B. brassicae* by residual/ leaf dip bioassay at different concentrations by ethanol extract of CCL, CIL and ATL.

Plants Extract	Conc. (mgmL^{-1})	Mean mortality		
		**24 h**	**48 h**	**72 h**
CCL	2.5	10±2.23^{d}	15±2.23^{d}	28.33±5.62^{d}
	5	13.33±1.29^{cd}	20±2.23^{cd}	31.67±5.62^{d}
	10	16.67±1.29^{c}	23.33±1.29^{c}	40±3.87^{d}
	15	18.33±1.29^{bc}	26.67±1.29^{c}	63.33±1.29^{c}
	20	23.33±2.58^{b}	40±2.23^{b}	76.66±1.29^{b}
	+ive control	81.67±1.29^{a}	88.33±1.29^{a}	98.33±3.87^{a}
	CK	0±0^{e}	0±0^{e}	1.67±1.29^{e}
	Statistics summary	*F*=163.97, *P*= 0.000<br>DF=104	*F*=151.28, *P*= 0.000<br>DF=104	*F*=53.93, *P*= 0.000<br>DF=104
CIL	2.5	6.67±2.58^{cd}	18.33±3.4^{d}	40±2.23^{d}
	5	8.33±1.29^{c}	30±2.23^{cd}	50±2.23^{c}
	10	13.33±1.29^{bc}	30±2.58^{cd}	56.67±4.40^{bc}
	15	16.67±3.41^{b}	38.33±2.58^{c}	61.67±2.58^{b}
	20	20±2.23^{b}	45±4.47^{c}	66.67±2.58^{b}
	+ive control	86.66±1.29^{a}	91.67±2.58^{b}	96.67±2.58^{a}
	CK	0±0^{e}	0±0^{e}	1.67±1.29^{e}
	Statistics summary	*F*=128.33, *P*= 0.000<br>DF=104	*F*=61.92, *P*= 0.000<br>DF=104	*F*=77.94, *P*= 0.000<br>DF=104
ATL	2.5	5±2.23^{de}	23.33±1.29^{d}	35±2.23^{e}
	5	15±2.23^{cd}	26.67±1.29^{d}	65±2.23^{d}
	10	18.33±2.5^{c}	43.33±1.89^{c}	76.67±1.29^{c}
	15	31.67±3.41^{b}	53.33±3.41^{b}	81.67±3.8^{c}
	20	35±3.83^{b}	60±2.23^{b}	88.33±1.29^{b}
	+ive control	81.67±3.41^{a}	90±3.87^{a}	96.6±1.29^{a}
	CK	0±0^{e}	0±0^{e}	1.67±1.29^{e}
	Statistics summary	*F*=57.34, *P*= 0.001<br>DF=104	*F*=97.45, *P*= 0.001<br>DF=104	*F*=261.85, *P*= 0.001<br>DF=104

Whereas: CCL (*C. colocynthis*); CIL (*C. Indica*); ATL (*A. argyi*); Values are represented as mean ± standard error followed by different superscripts are significantly different according to (Tukey's HSD $P > 0.05$).

Table S 2. Analysis of variance of the effect of mortality (%) versus treatment, concentration and time by residual/leaf dip assay

Source	Type III S.S	DF	M.S	*F*
C.M	177357.143	104	2860.599	101***
I	278876.190	1	278876.190	9851***
t	3342.857	2	1671.429	59.0***
C	128181.217	6	21363.536	754***
T	30891.270	2	15445.635	545***
t × C	2927.513	12	243.959	8.62***
t× T	820.635	4	205.159	7.25***
C ×T	9329.101	12	777.425	27.5***
t× C× T	1864.550	24	77.690	2.75***

Note: S.S (Sum of Square); M.S (Mean square); DF (Degree of freedom); C.M (Corrected model); I (Intercept); t (Treatments); C (Concentrations); T (Time); t×C (Treatments ×Concentrations); t×T (Treatments × Time); C×T (Concentrations × Time); t×C×T (Treatments × Concentrations × Time).

Table S 3. Mean mortality of *B. brassicae* by contact/aphid dip bioassay at different concentrations by ethanol extract of CCL, CIL and ATA.

Plants Extract	Conc. ($mgml^{-1}$)	Mean mortality		
		24 h	48 h	72 h
CCL	2.5	$53.33±5.16^{c}$	$56.67±6.83^{d}$	$58.33±5.62^{d}$
	5	$58.33±5.16^{c}$	$61.67±5.62^{cd}$	$65±4.47^{d}$
	10	$61.67±4.65^{bc}$	$66.67±3.41^{bcd}$	$71.67±±3.41^{cd}$
	15	$68.33±1.29^{bc}$	$75±2.23b^{c}$	$81.67±3.41^{bc}$
	20	$75±2.2^{b}$	$80±0.15^{ab}$	$83.33±2.58^{bc}$
	+ive control	$95±2.23^{a}$	$95±2.23^{a}$	$98.33±1.29^{b}$
	CK	$0±0^{d}$	$0±0^{e}$	$1.67±1.29^{e}$
	Statistics summary	*F*=33.18, *P*= 0.000 DF=104	*F*=34.90, *P*= 0.000 DF=104	*F*=41.62, *P*= 0.000 DF=104
CIL	2.5	$23.33±5.16^{d}$	$38.33±1.29^{c}$	$71.67±3.41^{c}$
	5	$33.33±5.16^{cd}$	$41.67±1.29^{c}$	$78.33±2.58^{bc}$
	10	$46.67±6.83^{bc}$	$56.67±1.29^{b}$	$78.33±2.58^{bc}$
	15	$55±2.23^{b}$	$60±2.23^{b}$	$81.67±1.29^{bc}$
	20	$58.33±2.58^{b}$	$63.33±2.58^{b}$	$81.67±1.29^{b}$
	+ive control	$91.67±2.58^{a}$	$96.67±2.58^{a}$	$98.33±1.29^{a}$
	CK	$0±0^{e}$	$0±0^{d}$	$1.67±1.29^{d}$
	Statistics summary	*F*=25.72, *P*= 0.000 DF=104	*F*=147.12, *P*= 0.000 DF=104	*F*=98.96, *P*= 0.000 DF=104
ATL	2.5	$36.66±2.58^{c}$	$43.33±2.58^{d}$	$46.67±3.41^{d}$
	5	$58.33±2.58^{bc}$	$56.67±3.41^{c}$	$60±4.47^{c}$
	10	$58.33±6.45^{ab}$	$61.67±1.29^{bc}$	$68.33±1.29^{c}$
	15	$60±5.91^{ab}$	$65±3.87^{bc}$	$83.33±1.29^{b}$
	20	$65±5.91^{ab}$	$70±2.23^{b}$	$93.33±3.41^{b}$
	+ive control	$88.33±3.41^{a}$	$93.33±3.41^{a}$	$100±0^{b}$
	CK	$0±0^{d}$	$0±0^{e}$	$1.67±1.29^{e}$
	Statistics summary	*F*=15.07, *P*= 0.000 DF=104	*F*=47.62, *P*= 0.000 DF=104	*F*=88.74, *P*= 0.000 DF=104

Whereas: CCL (*C. colocynthis*); CIL (*C. indica*); ATL (*A. argyi*); Values are represented as mean ± standard error followed by different superscripts are significantly different according to (Tukey's HSD $P > 0.05$)

Table S 4. Analysis of variance of the effect of mortality (%) versus treatment, concentration and time by contact/aphid dip assay.

Source	Type III S.S	D.F	M.S	*F*
C.M	158105.026	62	2550.081	44.4***
I	640211.640	1	640211.640	11152.0***
t	1683.598	2	841.799	14.6***
C	140995.767	6	23499.295	409***
T	7743.122	2	3871.561	67.4***
t × C	1086.772	12	90.564	1.57^{NS}
t × T	2311.640	4	577.910	10.1***
C × T	1821.693	12	151.808	2.64**
t × C × T	2462.434	24	102.601	1.79*

Note: S.S (Sum of Square); M.S (Mean square); C.M (Corrected model); I (Intercept); t (Treatments); C (Concentrations); T (Time); t×C (Treatments ×Concentrations); t×T (Treatments × Time); C×T (Concentrations × Time); t×C×T (Treatments × Concentrations × Time); N.S (non-significant)
